# The effect of a non-talking rule on the sound level and perception of patients in an outpatient infusion center

**DOI:** 10.1371/journal.pone.0212804

**Published:** 2019-02-28

**Authors:** Emma Zijlstra, Mariët Hagedoorn, Wim P. Krijnen, Cees P. van der Schans, Mark P. Mobach

**Affiliations:** 1 Hanze University of Applied Sciences, Groningen, The Netherlands; 2 Department of Health Psychology, University Medical Center Groningen, Groningen, The Netherlands; 3 University of Groningen, Groningen, The Netherlands; 4 Department of Rehabilitation Medicine, University Medical Center Groningen, Groningen, The Netherlands; 5 The Hague University of Applied Sciences, The Hague, The Netherlands; Medical University Graz, AUSTRIA

## Abstract

Noise is a common problem in hospitals, and it is known that social behavior can influence sound levels. The aim of this naturally-occurring field experiment was to assess the influence of a non-talking rule on the actual sound level and perception of patients in an outpatient infusion center. In a quasi-randomized trial two conditions were compared in real life. In the control condition, patients (n = 137) were allowed to talk to fellow patients and visitors during the treatment. In the intervention condition patients (n = 126) were requested not to talk to fellow patients and visitors during their treatment. This study measured the actual sound levels in dB(A) as well as patients’ preferences regarding sound and their perceptions of the physical environment, anxiety, and quality of health care. A linear-mixed-model showed a statistically significant, but rather small reduction of the non-talking rule on the actual sound level with an average of 1.1 dB(A). Half of the patients preferred a talking condition (57%), around one-third of the patients had no preference (36%), and 7% of the patients preferred a non-talking condition. Our results suggest that patients who preferred non-talking, perceived the environment more negatively compared to the majority of patients and perceived higher levels of anxiety. Results showed no significant effect of the experimental conditions on patient perceptions. In conclusion, a non-talking rule of conduct only minimally reduced the actual sound level and did not influence the perception of patients.

## Introduction

Patients visit an outpatient infusion center for treatments of various diseases, like cancer, vascular diseases, or muscle diseases. During therapy, some patients prefer a treatment environment to rest, whereas others prefer a social treatment environment with the opportunity to interact with fellow patients and visitors [1]. The social behavior of people in hospitals can influence the actual sound level and perception of patients [2]

Many studies showed that sound levels often exceed the WHO guideline of 35 dB(A) in a patient room [3–6]. High sound levels can cause noise-induced awakening, sleeplessness, and increased heart rates [7,8]. Noise can be defined as the presence of unwanted sound [9]. According to the WHO, the critical health effect for patients in a hospital treatment room is disturbance of rest and recovery [9]. A study at an intensive coronary ward showed that bad acoustics can even increase the hospital readmission rate and the need for additional intravenous beta-blockers [10].

In a hospital ward the most negatively perceived sounds were unnecessary sounds, for instance, cleaning machines, paging announcements, phones ringing, trolleys, and loud talking [2,11]. Nevertheless, patients reported human-related sounds most, like talking, laughing, and coughing [2,6]. The study of Baker et al. [8] showed that the maximum sound level was highest during conversation in a patient room (i.e., 67 dBA), with an average increase of 18 dB(A) during conversations.

Some patients may not be disturbed by these human-related sounds, while others may experience it as annoying [12]. According to Mackrill et al. [2]this difference in perception might depend on the individual coping method; some patients may become familiar with the sounds and they accept and habituate to sounds, while others may not be able to habituate to sounds and perceive it as disturbing. The perception of the sound of talking may also depend on the actual well-being of patients. Studies have shown that patients in oncology wards prefer to have the opportunity to choose between private and shared rooms [1,13], but when patients were able to interact they preferred shared rooms [13]. Sometimes patients may feel safe and secure when they hear others, while at other times they may be disturbed by these sounds and feel helpless because they cannot escape from the noise [14]. Therefore, in an outpatient infusion center the preferences of patients regarding sound may influence the individual perception of sound.

Quiet-time interventions may control the actual sound level by encouraging patients to rest and relax [15]. For example, quiet-time interventions in the afternoon (1.5 to 2 hours) reduced the sound levels with an average of 10 dB(A) in wards, and the mean sound level was correlated with the number of patients asleep and awake [16,17]. In contrast, an increase of 10 dB(A) is generally perceived as twice as loud [18]. However, these intervention studies manipulated multiple variables, such as a restriction of visitors, restriction of staff movements, promotion of closing doors, reduced light intensity, and lowered volume of technical equipment. Although relevant, for this reason, it is still unknown which individual element effectively reduced the sound level. Therefore, it is important to study specifically the influence of the sound of talking on the actual and perceived sound levels in a single intervention study.

Based on the ideas outlined above, we expect that a rule of conduct (i.e., a non-talking rule) reduces the actual sound level (hypothesis 1). Additionally, we expect an association between a rule of conduct and the patients’ perception, and it is expected that this association depends on the patients’ preferences (i.e., non-talking versus talking preference). Finally, we expect that the rule of conduct has more influence on patients with a clear preference as compared to patients with no preference. We hypothesize that patients with a preference for non-talking perceive less anxiety, proximity, crowdedness, and noise, and perceive more environmental satisfaction, privacy, pleasantness of the room, and satisfaction with healthcare treatment when there is a rule of conduct not to talk than when there is no rule of conduct (hypothesis 2). Conversely, patients with a preference for talking perceive less anxiety, proximity, crowdedness, and noise, and perceive more environmental satisfaction, privacy, pleasantness of the room, and satisfaction with healthcare treatment when there is no rule of conduct than when there is a rule of conduct not to talk.

## Methods

### Participants

Participants were recruited at the University Medical Center of Groningen (UMCG) between January 2015 and October 2015. Participants were outpatient adults visiting the outpatient infusion center, mostly for cancer treatments but also for treatments of chronic illnesses like Multiple Sclerosis, rheumatic disease, and Raynaud’s disease. Patients received different infusion treatments such as chemotherapy or other medicines, and bloodletting or blood transfusions. Eligible patients were 18 years or older, had visited the outpatient infusion center at least one time before, and had a minimum treatment duration of 30 minutes. Patients were excluded when they were not able to read and write Dutch.

A waiver for ethical assessment was provided by the Medical Ethical Committee of the Medical University of Groningen. The study was conducted according to the declaration of Helsinki. Written informed consent was obtained from participants during their visit at the outpatient infusion center.

### Study design

In a quasi-randomized trial, participants were assigned to one of the two conditions, namely no rule of conduct (i.e., talking condition) versus a rule of conduct (i.e., non-talking condition). Both conditions were carried out in the same treatment environment. Between January and October 2015 nine weeks were determined as measurement weeks. Patients who were scheduled to receive treatments during these weeks were included in the study. Be reminded that the study was room-based and included all patients in the treatment area in the described periods. The assignment to one of the two behavioral conditions occurred based on the scheduled appointments for a treatment. During three weeks, we assessed a group of 126 patients in the experimental weeks in which the non-talking rule was introduced. During six weeks, we assessed a group of 137 patients in the talking condition.

### Procedure

**Fig 1:**
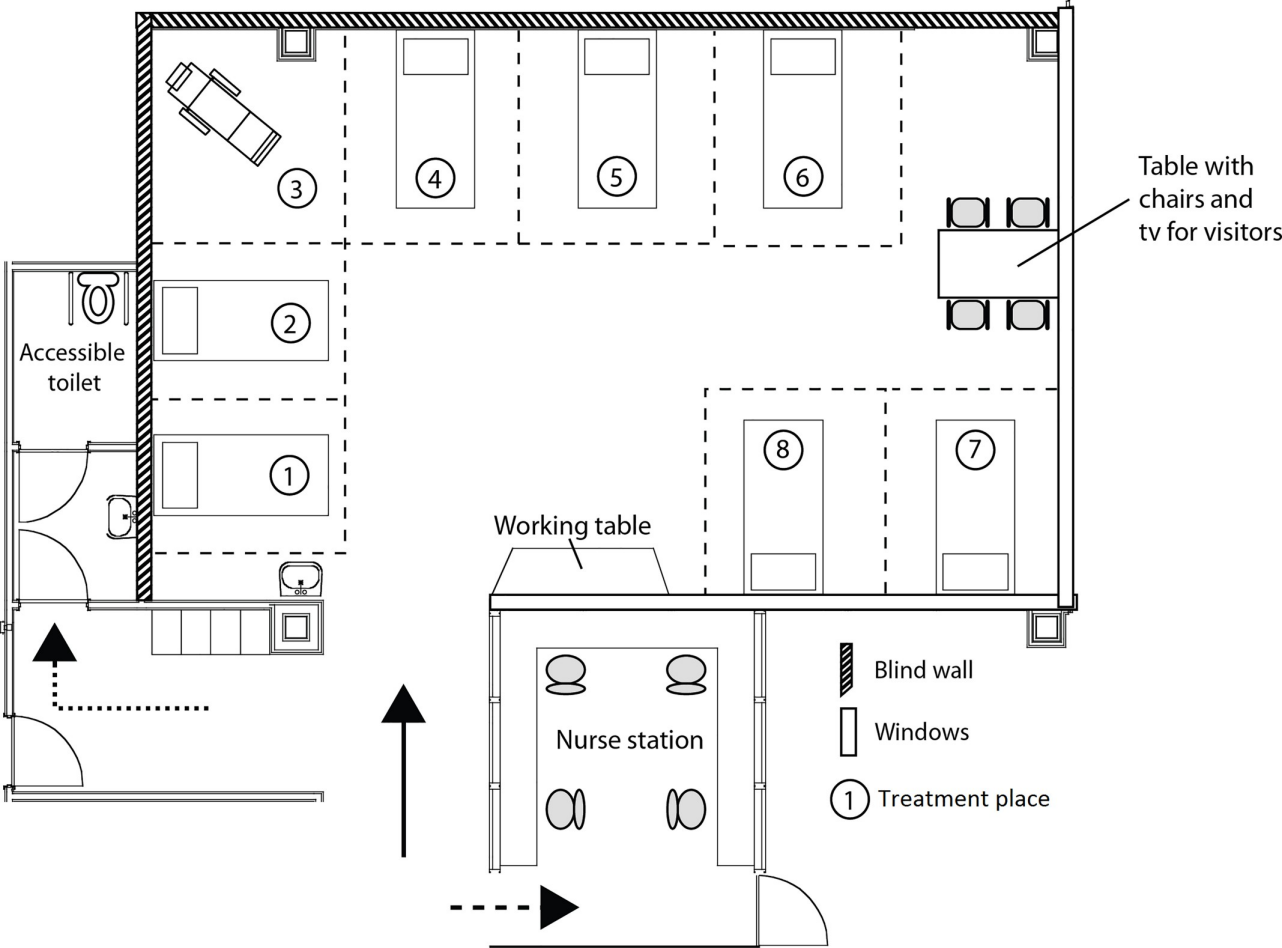
Map of the study site.

Before patients underwent their treatment at the outpatient infusion center, they received an appointment letter at their home address. Patients with an appointment in the experimental weeks received an additional information letter explaining the rule of conduct, one week before their appointment. By means of this letter, they were prepared for the rule of conduct not to talk to fellow patients and visitors during their treatment in order to respect the preferences of other patients, and also for the purpose of a sound environment test. At the day of the treatment, patients arrived at the reception and first took place in the waiting room. At the day of arrival in the experimental week, all patients were verbally reminded of the rule of conduct by the reception staff at the registration desk. A nurse picked up each patient from the waiting area and entered the treatment area (arrow, Fig 1). To test the effect of a non-talking rule and the applicability in a real-life setting, all patients in the treatment area were requested but not forced to comply with the rule. In this treatment area, patients received administration of medication via an injection or an intravenous line, or a blood treatment/bloodletting via an intravenous line. Therefore, an injection or needle was placed into the arm or hand of the patient. During the treatment patients took place on a treatment bed or chair (Fig 1). After 30 minutes of treatment, all eligible patients were asked by research assistants and nurses to fill in a questionnaire (S1 Appendix). The questionnaires were completed and handed in by patients during treatment at the outpatient infusion center.

### Study site

#### Architectural features

This study was carried out in a treatment area (94m^2^), which was secluded from other treatment rooms and areas. Consequently, patients were minimally disturbed by other patients or staff. Two sides of the area had blind walls and two sides of the area had windows (Fig 1). At one side of the area there were windows overlooking the main corridor of the hospital and perpendicular to this side there were windows with a view of the nurse station. This treatment area had a passage (no doors, arrow, Fig 1) to the corridor towards the waiting room. The entrance of the toilet for disabled patients was located in this corridor (dotted arrow, Fig 1) and the entrance of the nurse station (no doors, striped arrow, Fig 1).

#### Interior design features

**Fig 2:**
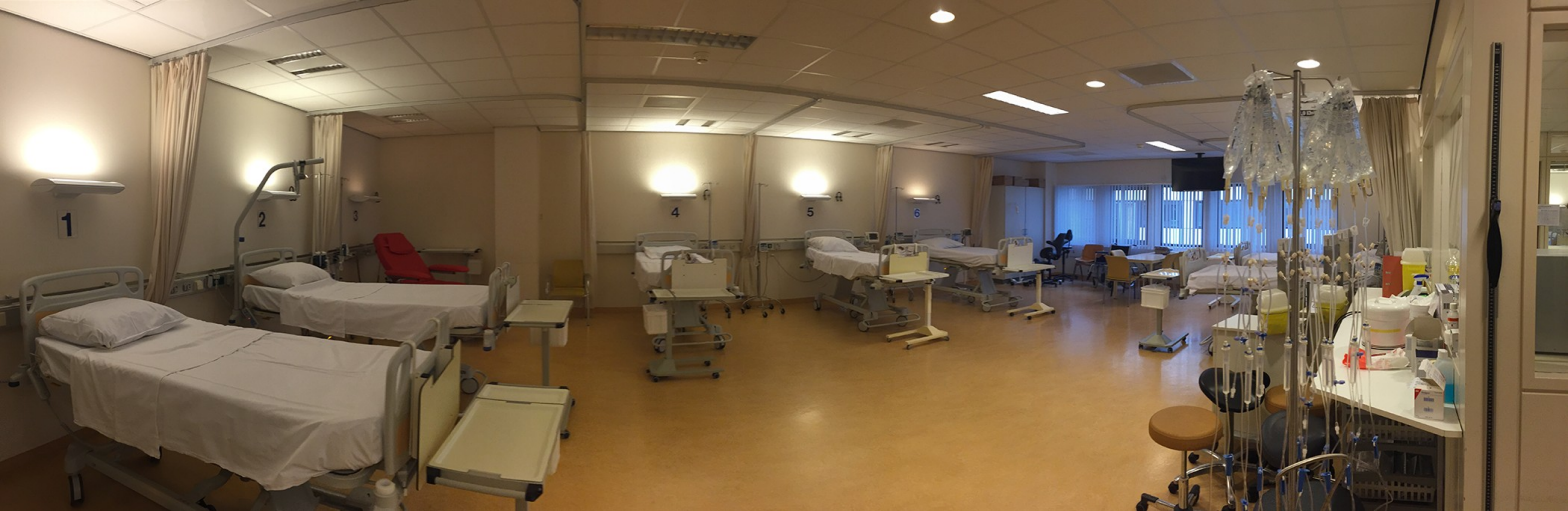
Impression outpatient infusion center.

An impression of the interior design features is shown in Fig 2. The treatment area included seven treatment beds and one treatment chair. A total of eight patients can be treated in this environment at the same time. A table with chairs was available for visitors (Fig 1). Moreover, a TV was placed on the ceiling (turned off in both conditions). For nursing staff a working table was placed against the wall of the nurse station. A clock was present above the window and working table, visible for some patients (places 1–6).

### Outcomes

#### Sound environment

The actual sound level in dB(A) in the treatment area was measured to assess actual differences in sound levels between the two study conditions. A-weighted decibels are a logarithmic unit to express the loudness of sound perceived by the human ear [18]. A sound level meter (Bruel & Kjaer 2250) was placed on the ceiling in the middle of the treatment area and measured the A-weighted equivalent levels (LAeq), minimum levels (LAFmin), and maximum levels (LAFmax) every minute during four days in each condition (Tuesday till Friday). In the non-talking condition, sound levels were measured between 13^th^ and 16^th^ of January, and in the talking condition between 20^th^ and 23^rd^ of January. Sound levels were measured between 10AM and 5PM representing the average sound level. During night time no treatment-related sounds were present at the outpatient infusion center, like patients, staff or alarm systems. To understand the sound levels that were generated by sources during the treatment, the average background sound level was measured during night between 12AM and 5AM as baseline.

#### Perceived anxiety

Perceived anxiety was measured by the Dutch version of the State-Trait Anxiety Inventory (STAI) of Spielberger [19]. On a 4-point Likert scale participants rate whether they felt calm, tense, upset, relaxed, content, or worried (1, not at all; 2, somewhat; 3, moderately; 4, very much). The sum of the 20-item state scale represents the level of state anxiety (i.e., how a person feels at that specific moment); higher scores indicate higher levels of anxiety (total score of 20 to 80). Cronbach’s alpha for the state-anxiety scale was .91.

#### Perceived environment

On a 7-point bipolar scale the perception of different environmental variables were measured based on five dimensions [20]. Each dimension consisted of one item and reflected (1) the satisfaction with the room (very dissatisfied versus very satisfied), (2) perceived privacy (not private versus private), (3) perceived proximity (too close to others versus too far from others), (4) perceived crowdedness (not crowded versus crowded), and (5) perceived noise (quiet versus noisy).

#### Perceived pleasantness of room

On a 7-point bipolar scale, participants rated the pleasantness of the room based on four dimensions (i.e., the environment seems: uncomfortable versus comfortable, drab versus colorful, boring versus interesting, and unattractive versus attractive) [20]. The scores of the four items were summed up, with higher scores reflecting a higher perception of pleasantness of the room. The range of scores is between 4 and 28. The scale showed high internal consistency with a Cronbach’s alpha .91.

#### Satisfaction with healthcare

Hawthorne et al. [21] developed a short questionnaire (7 items) to measure patient satisfaction with healthcare treatment based on seven dimensions (i.e., effectiveness, information, technical skill, participation, relationship, access and facilities, satisfaction general). The participants used a 5-point Likert scale ranging from (0) very dissatisfied to (4) very satisfied. The scores of the seven items were summed up; higher scores reflecting higher levels of satisfaction. The range of scores is between 0 and 28. Cronbach’s alpha for the satisfaction with healthcare scale was .67.

#### Patient preferences

Participants were asked (1 item) about their preferences for one of two types of treatment areas. Namely the non-talking room or the talking room. The non-talking room was defined as a treatment area where talking was not allowed, except with healthcare staff. The talking room was defined as a treatment area where it was allowed to speak to healthcare staff, but also, for instance, to fellow patients and visitors. The participants indicated a preference for either room or indicated that they had no preference for one or the other room.

### Data analysis

The main and interaction effects of a non-talking rule on the sound levels were examined by linear-mixed-modelling as it accounts for possible random effects during the day. The mixed-model included the minutes during the day as random effects, the measurement day as a fixed effect, and the fixed interaction effect between non-talking condition and measurement day. Standard errors were calculated using a restricted maximum likelihood approach.

To examine the perception of patients, two analyses were conducted. Firstly, a one-way MANOVA was conducted to examine the influence of a non-talking condition on the perceived anxiety, environmental satisfaction, privacy, proximity, crowdedness, noise, pleasantness of the room, and satisfaction with health care. A number of variables that may be related to the dependent variables were included to control for confounding effects, including gender, age, and diagnosis (i.e., cancer versus chronic illness). Secondly, a moderation analysis was conducted to test whether the relation between a non-talking condition and the dependent variables depends on the patients’ preference. The moderation analysis included the condition (talking versus non-talking condition) as independent variable and preference (talking preference versus non-talking preference versus no preference) as a moderator. We performed separate analyses for the dependent variables, namely, perceived anxiety, environmental satisfaction, privacy, proximity, crowdedness, noise, pleasantness of the room, and satisfaction with health care. Again, included confounding variables were gender, age, and diagnosis.

## Results

### Participants

In total, 263 patients participated in this study with a mean age of 53 years (SD = 14.33). From this group, 126 patients received their treatment in the non-talking condition and 137 patients in the talking condition. Half of the patients had a preference for talking (57%), 7% of the patients had a preference for non-talking, and 36% of the patients had no preference. The characteristics of the sample are presented in Table 1. Independent T-tests (ratio variables) and chi-square tests (nominal variables) were used to explore for differences between non-talking condition and talking condition.

**Table 1:** Study sample characteristics (N = 263).

	Non-talking condition (n = 126)	Talking condition (n = 137)	*p*
Male gender; N (%)	51 (41%)	55 (42%)	0.847^a^
Age; M (SD)	52.9 (14.7)	53.5 (14.1)	0.747^b^
Diagnosis; N (%)			0.092^a^
Cancer	84 (67%)	77 (57%)	
Chronic illness	41 (33%)	58 (43%)	
Preferences; N (%)			0.192^a^
Talking preference	73 (58%)	68 (56%)	
Non-talking preference	5 (4%)	12 (10%)	
No preference	47 (38%)	42 (34%)	

Note

^a^ Chi-square test

^b^ Independent T-test

### Rule of conduct and sound level

First, night measurements which were used as a baseline showed that the average sound level was 39.7 dB(A). Presented sound levels above 39.7 dB(A) were generated by sound sources during the treatment (e.g., sound of talking, sound of alarms). In the talking condition the mean sound level (LAeq) was 54.6 dB(A) (SD = 5.0) and in the non-talking condition the mean sound level was 51.9 dB(A) (SD = 4.7). The range of sound (LAFmin to LAFmax) in the talking condition was between 41 dB(A) and 69 dB(A), and in the non-talking condition between 40 dB(A) and 67 dB(A).

**Fig 3:**
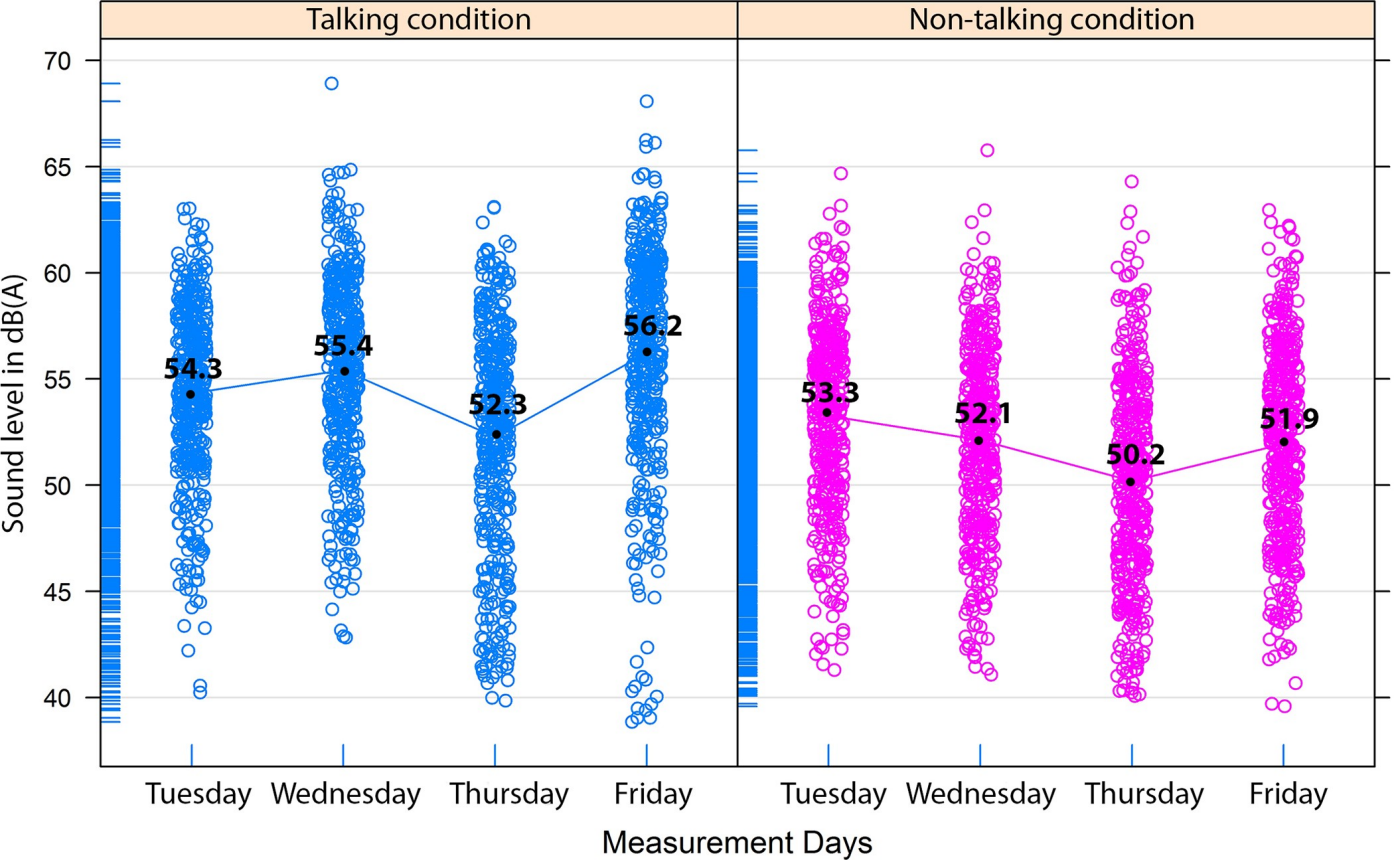
Average sound levels in dB(A) during four measurement days in each condition. Dots represent the sound level per minute.

Fig 3 shows that sound levels varied over days (Tuesday till Friday). Descriptive statistics of these measurement days (Table 2) showed fluctuations in the number of patients, treatment duration and occupancy rate. Since these variables (i.e., number of patients, treatment duration, and occupancy rate) were not independent they were excluded in the linear-mixed-model, but we controlled for measurement days. Results of linear-mixed-model (Table 3) confirmed our first hypothesis and showed a significant difference of 1.1 dB(A) in the mean sound level between the talking and non-talking condition, taken into account the effect of measurement days. The results of the linear-mixed-model showed a significant effect of measurement day on the average sound level. Moreover, results showed a significant interaction effect between the non-talking rule and measurement day. Therefore, also per day the average sound level (LAeq) in the non-talking condition was lower compared to the talking condition.

**Table 2:** Descriptive statistics sound levels in dB(A) during measurement days (non-talking condition versus talking condition).

	Non-talking condition				Talking condition			
	Number of patients	Cumulative duration treatments (in hours)	Occupancy rate	Sound level dB(A)	Number of patients	Cumulative duration treatments (in hours)	Occupancy rate	Sound level dB(A)
Tuesday	20	44.75	80%	53.3	18	41.50	74%	54.3
Wednesday	21	41.50	74%	52.1	20	43.00	77%	55.4
Thursday	13	43.25	77%	50.2	18	44.00	79%	52.3
Friday	18	36.00	64%	51.9	19	35.00	63%	56.2
Total week (mean)	18	41.38	74%	51.9	19	40.88	73%	54.6

Note: Be reminded that during the data collection of nine weeks the sound levels were measured during eight days equally divided over two weeks, allowing to test the main effect of condition in the linear-mixed-model.

**Table 3:** Results of the linear-mixed-model predicting the average sound levels in dB(A).

		Coef.	SE	T-value	*p*
	Main effects				
(Intercept)		54.324	0.228	238.627	*<* .*001*
Non-talking rule		-1.054	0.313	-3.3650	*<* .*001*
Tuesday^a^					
Wednesday		1.091	0.313	3.485	*<* .*001*
Thursday		-2.058	0.313	-6.572	*<* .*001*
Friday		1.897	0.313	6.058	*<* .*001*
	Interaction effects				
Non-talking rule * Wednesday		-2.246	0.443	-5.073	*<* .*001*
Non-talking rule * Thursday		-1.013	0.443	-2.289	.*022*
Non-talking rule * Friday		-3.227	0.443	-7.289	*<* .*001*

Coef. = Coefficient

^a^ Marks the reference category

### Rule of conduct and patient perception

The results of a one-way MANOVA (Table 4) showed that there was no significant main effect of the non-talking condition on the dependent variables level of perceived anxiety, environmental satisfaction, perceived privacy, perceived proximity, perceived crowdedness, perceived noise, perceived pleasantness of the room, and satisfaction with healthcare. In addition, results showed some significant effects of the covariates. Gender had a significant effect on pleasantness of the room, and age on environmental satisfaction, privacy, and pleasantness of the room. The covariate diagnosis showed a significant effect on satisfaction with healthcare.

**Table 4:** Results of one-way MANOVA of rule of conduct (non-talking versus talking) on perceived anxiety, environmental satisfaction, privacy, proximity, crowdedness, noise, pleasantness of room, and satisfaction with healthcare.

Independent variable	Dependent variable	F	*p*	Talking condition mean ± SD	Non-talking condition mean ± SD
Rule of conduct	Anxiety	0.087	0.768	32.3 ± 7.7	32.1 ± 8.9
	Environmental satisfaction	1.101	0.296	6.1 ± 1.1	6.2 ± 0.9
	Privacy	2.024	0.157	4.2 ± 1.6	4.5 ± 1.6
	Proximity	0.804	0.371	4.0 ± 1.1	4.7 ± 5.9
	Crowdedness	0.733	0.393	4.3 ± 0.9	4.1 ± 0.8
	Noise	0.000	0.990	4.2 ± 1.2	4.2 ± 4.7
	Pleasantness of room	1.149	0.285	16.5 ± 5.4	16.8 ± 5.5
	Satisfaction with healthcare	0.463	0.497	29.8 ± 2.9	29.7 ± 2.5
Covariates					
Gender^a^	Anxiety	1.292	0.257		
	Environmental satisfaction	0.121	0.728		
	Privacy	2.507	0.115		
	Proximity	0.113	0.737		
	Crowdedness	0.735	0.392		
	Noise	1.519	0.220		
	Pleasantness of room	4.972	*0*.*027*		
	Satisfaction with healthcare	1.795	0.182		
Age	Anxiety	2.277	0.133		
	Environmental satisfaction	10.082	*0*.*002*		
	Privacy	7.871	*0*.*006*		
	Proximity	0.754	0.387		
	Crowdedness	0.007	0.932		
	Noise	0.004	0.952		
	Pleasantness of room	23.357	*0*.*000*		
	Satisfaction with healthcare	0.066	0.797		
Diagnosis^b^	Anxiety	3.087	0.081		
	Environmental satisfaction	0.634	0.427		
	Privacy	0.032	0.858		
	Proximity	0.444	0.506		
	Crowdedness	0.327	0.568		
	Noise	0.030	0.863		
	Pleasantness of room	1.503	0.222		
	Satisfaction with healthcare	3.973	0.048		

Note:

^a^ Female versus male (male is reference category)

^b^ Chronic illness versus cancer (cancer is reference category)

According to our second hypothesis we expected an interaction between condition and preference. However, our descriptive results (Table 1) showed that the number of participants who preferred non-talking was too small to test meaningful statistical differences. Therefore, only a moderation analysis was conducted for the patients who preferred talking and had no preference (Table 5). The results of the moderation analysis showed no significant interaction effect. Hence, the relation between the condition (talking versus non-talking) and the dependent variables did not depend on the patients’ preferences (talking versus no preference). However, results did show a significant effect of preference on how patients rated the pleasantness of the room (p = .038). Patients who preferred talking rated the room as less pleasant compared to patients without a preference, regardless of the condition.

**Table 5:** Results interaction analysis between condition (talking versus non-talking) and patient preference (no preference versus talking preference).

	Condition^a^				Preference^b^				Condition * preference			
	*B*	SE	t	*p*	*B*	SE	t	*p*	*B*	SE	t	*p*
Anxiety	-.91	1.25	-.72	.470	-.73	1.30	-.56	.576	1.28	2.55	.50	.616
Environmental satisfaction	.17	.13	1.32	.188	-.07	.14	-.48	.629	.32	.27	1.19	.237
Privacy	.33	.22	1.51	.134	.34	.23	1.49	.138	.82	.45	1.84	.067
Proximity	.56	.52	1.07	.285	.31	.54	.57	.568	1.29	1.06	1.22	.225
Crowding	-.14	.12	-1.15	.251	-.12	.13	-.92	.359	.25	.25	1.01	.312
Noise	-.07	.43	-.16	.872	-.12	.45	-.45	.656	.84	.87	.96	.338
Pleasantness of room	.27	.68	.39	.350	-1.49	.72	-2.08	.*038*	1.31	1.40	.94	.350
Satisfaction with healthcare	.02	.39	.05	.961	.01	.41	.01	.996	.70	.80	.87	.386

Included covariates: gender, age and diagnosis

^a^ Talking condition versus non-talking condition (non-talking condition is reference category)

^b^ Talking preference versus no preference (no preference is reference category)

Note: Non-talking preference sample was too small to test statistical analyses.

In addition, the differences in descriptive means were explored to gain additional insight in the three different preferences of patients (i.e., non-talking preference, talking preference, no preference). Results showed that patients with a non-talking preference revealed higher levels of perceived anxiety compared to patients with a talking preference and no preference (Table 6). In addition, results of patients that preferred non-talking showed the lowest score on environmental satisfaction, perceived privacy (i.e. not private), and perceived proximity (i.e. too close to others), and perceived the room as less pleasant compared to patients who preferred talking or had no preference. In addition, patients with non-talking preference perceived more crowding and noise compared to patients with a talking preference and no preference. Scores of satisfaction with healthcare did not differ considerably between the groups.

**Table 6:** Mean and standard deviations of dependent variables per preference.

Dependent variable	Non-talking preference (n = 17)	Talking preference (n = 141)	No preference (n = 89)
Anxiety	37.9 ± 7.9	31.8 ± 16.4	32.1 ± 18.0
Environmental satisfaction	5.4 ± 1.4	6.2 ± 1.0	6.3 ± 0.9
Privacy	2.8 ± 1.8	4.5 ± 1.6	4.2 ± 1.7
Proximity	3.3 ± 1.9	4.4 ± 4.6	4.0 ± 3.6
Crowdedness	5.0 ± 1.2	4.2 ± 0.8	4.3 ± 0.9
Noise	4.8 ± 1.7	4.2 ± 3.7	4.2 ± 2.9
Pleasantness of room	13.5 ± 4.0	16.4 ± 5.3	18.0 ± 5.1
Satisfaction with healthcare	29.6 ± 2.6	29.9 ± 2.6	29.9 ± 2.8

Note: Number of patients who preferred a non-talking condition was too small to test statistical differences.

There were 16 missing values in preference, because these patients did not indicate their preference.

## Discussion

The results of this study showed that a rule of conduct (i.e., request for patients not to talk to fellow patients and visitors) reduced the sound level in an outpatient infusion center. The observed differences were very small, but statistically significant. In addition, the rule of conduct did not significantly influence the perception of patients, neither positive nor negative. The minority of patients preferred a non-talking condition in the outpatient infusion center, and the results showed that this group of patients perceived higher levels of anxiety and perceived the outpatient infusion center as less positive, compared to patients who preferred talking or had no preference. However, due to limitations in the sample size the relevant and exciting findings at the non-talking preference group need further investigation in future research to allow more robust conclusions.

First, in this study, the average sound level was 52 dB(A) in the non-talking condition and 55 dB(A) in the talking condition, with a maximum sound level of 69 dB(A). The average sound level in the control condition was 19.6 dB(A) higher than the recommended level of 35 dB(A) in hospital treatment rooms stated in the WHO guideline. Furthermore, in the non-talking condition the minimum sound level was 40 dB(A) and still exceeded the recommended level. This minimum sound level of 40 dB(A) can be compared with the sound of whispering (Harris, 1979). People can speak with a relaxed voice when sound levels are below 50 dB(A), sound level of 57 dB(A) can be compared with a with a normal voice, 65 dB(A) with a raised voice, 74 dB(A) with a very loud voice, and 82 dB(A) with a shouting voice [22]. Therefore, in the current outpatient infusion center, with a maximum sound level 69 dB(A), people may need to raise their voice in conversations [22].

Second, this study showed a statistically significant, but rather small effect of a non-talking rule on the actual sound level with an average of 1.1 dB(A). Results showed a significant interaction effect between the non-talking condition and measurement day, and showed a reduction in sound level up to 3.3 dB(A) on Wednesday, up to 2.1 dB(A) on Thursday, and up to 4.2 dB(A) on Friday. A reduction of 3 dB(A) is a halving of sound sources (i.e., acoustic power). Other studies [16,17] showed a reduction of 10 dB(A) through a quiet-time intervention. So, the observed differences in this current study were rather small. However, the other studies included multiple manipulations (patient behavior, staff behavior, technical equipment). Be reminded that our intervention study only manipulated patient behavior by means of a rule of conduct, namely non-talking. According to the focus theory of normative conduct [23], humans behave according to descriptive norms (typical or normal behavior) and injunctive norms (rules or beliefs). The reduced sound level suggests that the typical behavior of talking in the outpatient infusion center (descriptive norm) can be changed by setting a non-talking rule of conduct (injunctive norm).

Third, in contrast with the results of other studies [15,16], our intervention study showed no influence of the non-talking rule on the perception of patients. Previous studies were conducted in wards where patients stayed overnight. This may be explained by the relatively small average reduction of 1 dB(A) in our study compared to an average reduction of 10 dB(A) in the quiet-time intervention study including multiple manipulations [16]. On the other hand, patients in outpatient infusion centers may experience their visit differently compared to inpatients, because they perceive the benefit of leaving after the treatment is finished [24]. Therefore, patients may accept the sound as a part of being in a hospital [2], and may adapt to the current situation and pay less attention to the noise [12].

Fourth, one of our concerns was that the noise not related to talking was quite high, and therefore the non-talking rule didn’t have such an impact on overall noise reduction. This can be explained by sounds due to conversations between nurses and patients about the patients’ health and treatment. In addition, other studies also showed that the effect of technical related sounds were mentioned by patients [2]. Studies showed that the increasing use of medical device alarms cause noise [25]. At this outpatient infusion center acoustic alarms were used as a warning system for nurses. Since most patients underwent infusion therapy and received medication via an intravenous line, each individual patient had a medical alarm system standing next to their bed or chair which can be explained as a reason that the overall sound level was pretty high.

Fifth, this study showed that more than half of the patients preferred a talking condition, around one-third of the patients had no preference, and only a minority preferred a non-talking condition. Despite the small group of patients who preferred a non-talking condition, results suggest that they had different perceptions compared to other preferences. This group of patients perceived more anxiety, lower satisfaction levels, perceived the room as less pleasant, perceived less privacy, and felt close proximity (too close to others). Dijkstra et al. [26] suggested that the perception of a stimulus (e.g., sound) depends on the characteristics of the patient population. Our results suggest that it may specifically depend on the individual preference of patients and that patients who prefer non-talking perceive the current outpatient infusion center (non-talking or talking condition) more negative compared to patients who prefer talking or had no preference. However, due to a small group who preferred a non-talking condition, further research in a larger sample is necessary to test whether the differences in percentages also reflects a true statistically significant difference.

In conclusion, our study showed that a rule of conduct seems to influence patient behaviors in a field-setting, but by doing so, only slightly reduces the actual sound level, however not to an impactful level in an outpatient infusion center. The well-being and perception of patients was not influenced by the rule of conduct. However, our results suggest that patients who preferred non-talking (although having limitations due to a small sample size and related statistical power), perceived the environment more negatively compared to the majority of patients and perceived higher levels of anxiety. The results indicate that a rule of conduct is not sufficient to reduce sound level and improve the perceptions of patients in an outpatient infusion center. Patients in an outpatient infusion center might potentially benefit from a patient-centered spatial design where they have the opportunity to choose whether to rest in silence or to communicate with others

## Further research and limitations

This study has some limitations. First, patients with different preferences were assigned to a non-talking and talking condition. It is expected that the average sound level will decrease more when exclusively patients with a non-talking preference receive their treatment in a non-talking condition. Therefore, it is expected that the opportunity to choose between a non-talking and talking condition may potentially influence the perception of patients positively. Further research should clarify whether the opportunity to choose between conditions, for instance, with a spatially-targeted planning system, shows a larger influence on the sound level and, in addition, a positive influence on the perception of patients.

Second, our results showed a significant interaction effect between the non-talking rule and measurement days on sound levels. However, due to the complexity of the naturally occurring field experiment and to financial limitations, the sound measurements were limited to eight days and we did not measure sound sources and sound levels of treatment equipment separately. Results of the descriptive statistics of the measurement days showed fluctuations in the number of patients, occupancy rate and sound levels (e.g., lower occupancy rate on Friday but an increase in sound level). These fluctuations cannot be explained with the data of the current study design. Further research is necessary to distinguish and unravel the causes (sound sources) of the sound levels.

Third, patients were only included in this study when they visited the outpatient infusion center at least for the second time. Therefore, patients in the talking condition (control condition) may have adapted to the current situation and were used to the sound level. It is expected that patients visiting the outpatient infusion center for the first time may have different preferences and perceive the sound environment more negative compared to recurring patients. Further research is necessary to examine whether the preferences and perceptions differ between recurring patients and patients who visit an outpatient infusion center for the first time.

Finally, the results of this study showed a difference between preferences and the perception of patients. However, we cannot generalize the results because the group of patients who preferred non-talking was a relatively small group. Further qualitative research is necessary to provide a rich understanding of the experiences of patients with different preferences. Gaining a greater understanding of the underlying feelings and reasons of patients visiting an outpatient infusion center and how this would contribute to a better understanding of how to improve the experiences and well-being of patients in an outpatient infusion center.

## Supporting information

S1 Appendix Questionnaire. (DOCX)

S1 Dataset Dataset sound level. (XLSX)

S2 Dataset Dataset questionnaire. (XLSX)
