# Supplementary material for: The effect of a non-talking rule on the sound level and perception of patients in an outpatient infusion center
Source: PLoS One. 2019 Feb 28;14(2):e0212804. doi: 10.1371/journal.pone.0212804 (PMC6395026; doi:10.1371/journal.pone.0212804)
Supplement: S1 Appendix — (DOCX) [file pone.0212804.s001.docx]

# S1 Appendix. Questionnaire

| Scale / dimension | Question | Answer type: |
| --- | --- | --- |
| Gender | 1. I am: | Male / Female |
| Age | 1. My age is: | Years |
| Perceived anxiety | 1. I feel calm 2. I feel secure 3. I am tense 4. I feel regretful 5. I feel at ease 6. I feel upset 7. I am presently worrying over possible misfortunes 8. I feel satisfied 9. I am anxious 10. I feel comfortable 11. I feel self-confident 12. I feel nervous 13. I feel jittery 14. I feel indecisive 15. I am relaxed 16. I feel content 17. I am worried 18. I feel over-excited and rattled 19. I feel joyful 20. I feel pleasant | 4-point Likert scale:   - Not at all - Somewhat - Moderately - Very much |
| Environmental satisfaction | 1. How satisfied are you with the treatment environment of this outpatient infusion center? | 7-point bipolar scale:  Very dissatisfied (1) vs Very Satisfied (7) |
| Privacy | 1. Rate the treatment environment… | 7-point bipolar scale:  Not private (1) vs. Private (7) |
| Proximity | 1. Rate the treatment environment… | 7-point bipolar scale:  Too close to other patients (1) vs. Too far from other patients (7) |
| Crowdedness | 1. Rate the treatment environment… | 7-point bipolar scale:  Not crowded (1) vs. Crowded (7) |
| Noise | 1. Rate the treatment environment… | 7-point bipolar scale:  Quiet (1) vs. Noise (7) |
| Perceived pleasantness of the room | 1. Please indicate how the treatment environment seems: | 7-point bipolar scale  Uncomfortable (1) vs. Comfortable (7) |
| Perceived pleasantness of the room | 1. Please indicate how the treatment environment seems: | 7-point bipolar scale  Drab (1) vs. Colorful (7) |
| Perceived pleasantness of the room | 1. Please indicate how the treatment environment seems: | 7-point bipolar scale  Boring (1) vs. Interesting (7) |
| Perceived pleasantness of the room | 1. Please indicate how the treatment environment seems: | 7-point bipolar scale  Unattractive (1) vs. Attractive (7) |
| Satisfaction with healthcare | 1. How satisfied are you with the effect of your treatment? | 5-point Likert scale   - Very satisfied - Satisfied - Neither satisfied nor dissatisfied - Dissatisfied - Very Dissatisfied |
| Satisfaction with healthcare | 1. How satisfied are you with the explanations the health professional has given you about the results of your treatment? | 5-point Likert scale   - Very satisfied - Satisfied - Neither satisfied nor dissatisfied - Dissatisfied - Very Dissatisfied |
| Satisfaction with healthcare | 1. The health professional was very careful to check everything when examining you. | 5-point Likert scale   - Strongly agree - Agree - Not sure - Disagree - Strongly disagree |
| Satisfaction with healthcare | 1. How satisfied were you with the choices you had in decisions affecting your health care? | 5-point Likert scale   - Very satisfied - Satisfied - Neither satisfied nor dissatisfied - Dissatisfied - Very Dissatisfied |
| Satisfaction with healthcare | 1. How much of the time did you feel respected by the health professional? | 5-point Likert scale   - All of the time - Most of the time - About half the time - Some of the time - None of the time |
| Satisfaction with healthcare | 1. The time you had with the health professional was too short. | 5-point Likert scale   - Strongly agree - Agree - Not sure - Disagree - Strongly disagree |
| Satisfaction with healthcare | 1. Are you satisfied with the care you received in the outpatient infusion center? | 5-point Likert scale   - Very satisfied - Satisfied - Neither satisfied nor dissatisfied - Dissatisfied - Very Dissatisfied |
| Preference | 1. If you had the opportunity, which treatment environment would you prefer to receive the next treatment? | Non talking room / Talking room / No preference for either room |
